# Forty-Three-Year-Old Female with Dopamine Secreting Pheochromocytoma of the Adrenal Gland

**DOI:** 10.1155/2017/1736326

**Published:** 2017-08-31

**Authors:** Tyler Haden, Marcin Zuberek, Naveen Pokala

**Affiliations:** Division of Urology, University of Missouri School of Medicine, One Hospital Dr, Columbia, MO 65212, USA

## Abstract

We report on a 43-year-old, asymptomatic female who presented with incidental finding of left adrenal mass. MRI gave concerns for possible pheochromocytoma but markers for pheochromocytoma were not elevated as expected. 24-hour urine dopamine levels (6988 *μ*g/day) were significantly elevated. The patient successfully underwent robotic assisted radical left adrenalectomy and was diagnosed with a dopamine secreting pheochromocytoma. Pathology revealed increased malignant potential associated with the tumor. The patient underwent full metastatic workup, which was negative. At two years of follow-up there was no recurrence and normalization of lab values.

## 1. Introduction

**Figure 1:**
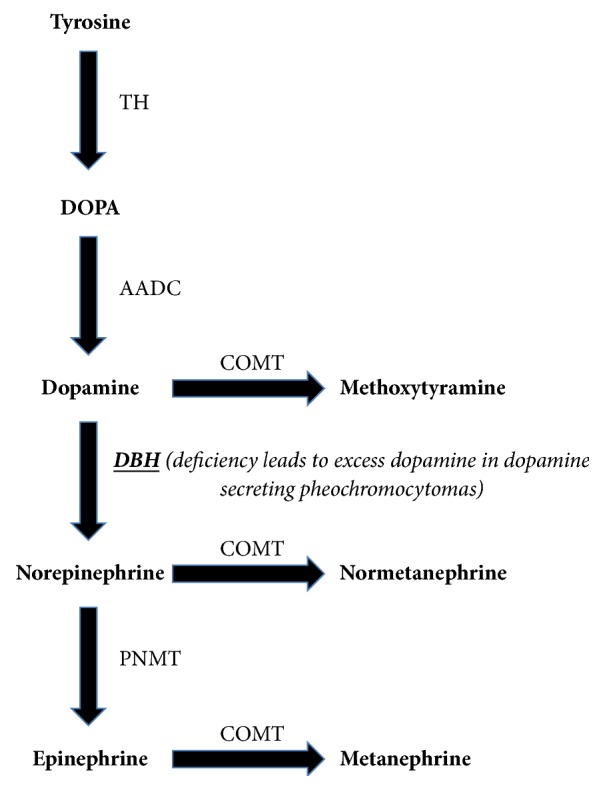
Catecholamine synthesis pathway. TH: tyrosine hydroxylase; AADC: aromatic amino acid decarboxylase; DBH: dopamine B-hydroxylase; PNMT: phenylethanolamine-*N*-methyltransferase; COMT: catechol-*O*-methyltransferase.* Deficiency in dopamine B-hydroxylase leads to elevated dopamine levels in dopamine secreting pheochromocytomas*.

Pheochromocytomas or paragangliomas are neuroendocrine tumors that arise from chromaffin cells. The annual incidence of pheochromocytoma/paragangliomas is approximately 0.8/100,000 person-years [1]. The classic symptoms consist of episodic headache, sweating, and tachycardia [2] but may be present in only 50% of patients and are usually paroxysmal. Classic symptoms are due to excess adrenaline and/or noradrenaline production. Initial testing usually begins with plasma-free metanephrines or 24-hour urinary fractionated metanephrines [3]. Functional pheochromocytomas that produce dopamine primarily or exclusively are extremely rare. Poirier et al. [4] reported 35 abdominal dopamine secreting pheochromocytomas or “dopaminomas” since 1980 in the literature. The elevated dopamine level in these tumors is caused by a deficiency in dopamine-beta-hydroxylase, which converts dopamine to norepinephrine [5].  Figure 1 shows the catecholamine synthesis pathway. Kiernan and Solórzano [3] report plasma or urinary dopamine and its metabolite (methoxytyramine) can be elevated in pheochromocytomas but are not routine for diagnostic purposes.

## 2. Case Report

A 43-year-old Hispanic woman was referred to our urology clinic at our institution for an incidental finding of left sided adrenal mass on computed tomography (CT) done for right hip and abdominal pain. CT revealed a 5.8 cm enhancing mass from the left adrenal gland. Confirmatory MRI revealed a 6 cm left adrenal mass (Figure 1). The patient was asymptomatic with no history that would suggest increased catecholamines.

Patient was referred to endocrinology for further workup for possible pheochromocytoma. 24-hour urine metanephrines 220 mcmol/mol (0–172) and normetanephrines 276 mcmol/mol (0–247), as well as plasma normetanephrines 1.15 nmol/L (0.00–0.89) and metanephrines .53 nmol/L (0.00–0.49), were all mildly elevated. Urine epinephrine and norepinephrine were normal. 24-hour urine dopamine was drawn due to minimal elevation of metanephrines and normal catecholamines. Dopamine levels were severely elevated, >25x normal range at 6988 *μ*g/day (0–250). Surgical intervention was recommended to the patient likely for a dopamine producing pheochromocytoma (dopaminoma). The patient elected to proceed with robotic assisted left radical adrenalectomy. Preoperatively patient was placed on alpha blockade with phenoxybenzamine. Intraoperative and postoperatively patient had no issues with blood pressure control. There were no postoperative complications.

Gross pathology revealed 4 × 4 × 3 cm black partially encapsulated mass attached to adrenal gland. Tumor was revealed to be a pheochromocytoma with staining pattern positive for chromogranin, synaptophysin, and neuron specific enolase supporting the diagnosis of pheochromocytoma. The tumor had focal extension through capsule into surrounding adipose tissue along with focal vascular invasion. Tumor was assigned a Pheochromocytoma of the Adrenal Gland Scaled Score (PASS) score of 4 (two points for extension into adipose tissue, one point for vascular invasion, and one point for capsular invasion) increasing risk of malignant potential. The mitoses were less than three per high power fields, and no atypical mitotic figures were noted. The patient was diagnosed with a dopamine secreting pheochromocytoma.

After mass resection, 24-hour plasma and urine dopamine, normetanephrines, and metanephrines were repeated at 1 and 6 months and were normal. Urinary dopamine decreased from 6988 *μ*g/day preoperatively to 180 *μ*g/day (0–250) postoperatively. Normetanephrines and metanephrines did not have a significant change. All labs remained within normal levels at follow-up two years postoperatively. Due to PASS score of 4, (PET) scans were obtained at 6 months and two years postoperatively with no signs of disease. Metaiodobenzylguanidine (MIBG) scan was also done postoperatively with no uptake noted. Genetic testing for multiple endocrine neoplasia type 2, neurofibromatosis type 1, and von Hippel Lindau were negative.

## 3. Discussion

Dopamine secreting pheochromocytomas are extremely rare, as only 35 abdominal dopaminomas have been reported in the literature in 35 years. [4]. Poirier et al. also report that there have been only 10 reported exclusively dopamine secreting abdominal pheochromocytomas, of which only 1 was benign. Our case's pathology revealed a PASS score of 4. Studies have shown a PASS of ≥4 for pheochromocytomas have a greater risk of malignant potential [6]. The elevated PASS score along with known aggressive behavior of dopaminomas prompted for further evaluation. MIBG is considered the best tool to evaluate for the presence of a neuroendocrine tumor and metastases [7], but not all dopamine-excreting tumors accumulate MIBG. For this reason a PET scan may be used in conjunction with MIBG. Our patient has had no signs of disease reoccurrence at two years of follow-up.

Another issue with dopamine secreting pheochromocytomas is difficulty in diagnosis. Many institutions do not routinely screen for dopamine when doing urinary or plasma catecholamine screening [3, 4]. This can lead to missing elevated dopamine levels associated with dopaminomas. Also dopamine levels can be elevated for a variety of medical conditions. The third issue with diagnosing dopaminomas is that many cases do not present with typical paroxysmal symptoms associated with pheochromocytomas such as headache, sweating, and hypertension [2]. Our patient had no classic paroxysmal symptoms and normal blood pressures. The initial workup started after patient had incidental finding of left adrenal mass.

Historical management for a classic pheochromocytoma is preoperative *α*- and intraoperative *β*-blockade to prevent life-threatening hypertensive crises. Alpha-blocker treatment has been considered contraindicated for dopamine secreting pheochromocytomas because case reports have shown it can lead to cardiovascular collapse and potential hypotensive crisis [8]. This action is thought to be caused by two possible mechanisms. Elevated dopamine levels in dopaminomas are believed to be caused by a deficiency in dopamine-beta-hydroxylase, which converts dopamine to norepinephrine [5]. This deficiency in norepinephrine may contribute to normal blood pressures associated with dopamine secreting pheochromocytomas. Also dopamine has no effect on *α*-receptors but acts on D1 and D2 receptors. In our case urinary dopamine was used as a surrogate for plasma dopamine preoperatively due to extreme elevation of urinary dopamine. Urinary dopamine is not a direct correlation to circulating plasma dopamine but previous studies looking at dopamine secreting paragangliomas have shown urinary outputs of dopamine were positively related to plasma concentrations of dopamine [9]. Our patient did receive preoperative alpha blockade with phenoxybenzamine with no cardiovascular or blood pressure issues noted pre- or postoperatively. Tam et al. [10] have also reported on a patient receiving phenoxybenzamine and propranolol preoperatively without any complications. Definitive treatment of any pheochromocytoma is surgical excision. Studies also have shown laparoscopic resection of large adrenal tumors to be safe [11]. The resection of our dopamine secreting pheochromocytoma was completed with robotic assisted laparoscopic adrenalectomy without complication.

Dopamine secreting pheochromocytomas are rare. These tumors have increased risk of malignant potential and may be difficult to diagnose due to atypical symptoms and lack of routine screening for dopamine. Robotic assisted adrenalectomy is an acceptable method of tumor excision in appropriately selected patients.
